# Emotional Health Work of Women With Female Genital Cutting Prior to Reproductive Health Care Encounters

**DOI:** 10.1177/10497323211049225

**Published:** 2021-12-06

**Authors:** Danielle Jacobson, Daniel Grace, Janice Boddy, Gillian Einstein

**Affiliations:** 1 Dalla Lana School of Public Health, 274071 University of Toronto, Toronto, ON, Canada; 2 Department of Anthropology, 7938 University of Toronto, Toronto, ON, Canada; 3 Department of Psychology, 7938 University of Toronto, Toronto, ON, Canada; 4 Department of Gender Studies, Linköping University, Linköping, Sweden

**Keywords:** minorities, cultural competence, emotion work, women’s health, gender, health promotion, users’ experiences, health care

## Abstract

We used institutional ethnography to explore the social relations that shaped the reproductive health care experiences of women with female genital cutting. Interviews with eight women revealed that they engaged in discourse that opposed the practices of cutting female genitals as a human-rights violation. This discourse worked to protect those affected by the practices, but also stigmatized female genital cutting, making participants anticipate experiencing stigmatization during health care. Women’s engagement in this discourse shaped their emotional health work to prepare for such encounters. This work included navigating feelings of worry, shame, and courage to understand what to expect during their own appointment; learning from family/friends’ experiences; and seeking a clinic with the reputation of best care for women with female genital cutting. It is important to strive for more inclusive health care in which women do not have to engage in emotional health work to prepare for their clinical encounters.

## The Current Study

This study is part of a larger institutional ethnographic project focusing on the research question: What are the social relations that shape the reproductive health care experiences of women with female genital cutting in Toronto, Canada? Female genital cutting involves removing parts of the external genitalia of those assigned female at birth for non-medical reasons. The World Health Organization (2008) categorizes the practices into four types: type I (removing the clitoris); type II (removing the labia minora); type III (cutting/stitching together the labia minora [IIIa] and/or majora [IIIb], leaving a small opening); and type IV (all other harmful procedures). Female genital cutting is practiced in many parts of Africa, Asia, and the Middle East (World Health Organization, 2018). Reasons for these practices depend on the cultural context (Einstein et al., 2018) and include enhancing attractiveness (Boddy, 1982), hygiene (Hearst & Molnar, 2013), and marriage prospects (Abathun et al., 2016).

Across the larger project, the objective was to link women’s accounts of their health care experiences with what happened at the institutional level (i.e., the social processes that relate to the care of reproductive health within Toronto’s health care institution). This linking up helped us to understand the social relations that shaped women’s experiences within the reproductive health care context. Social relations go beyond relationships between people, instead drawing attention to connections between people, what they do, and written/unwritten texts.

Each portion of the larger study centered in on a particular component of this set of social relations. The current study focused on a particular discourse that opposed female genital cutting that we refer to as “anti-female genital mutilation discourse.” “Discourse” refers to the particular set of written/unwritten ideas and conceptual knowledge communicated through verbal language and text. “Anti-female genital mutilation” refers to the opposition to cutting the genitals of those assigned female at birth (this is described further below). The objective of the current study was to explore how anti-female genital mutilation discourse, as a particular social relation, shapes the reproductive health care experiences of women with female genital cutting, including their work to prepare for the care encounter.

### Literature Review

Research shows that women with female genital cutting receiving reproductive health care in western countries often have problematic delivery outcomes (Berggren et al., 2006; Johnson et al., 2005); these include an increased likelihood, compared to women without female genital cutting, for episiotomy and perineal trauma (Belihu et al., 2017). These (i.e., episiotomy; Moxey & Jones, 2016) and other obstetrical procedures (i.e., caesarean section; Ameresekere et al., 2011) are often feared by women with female genital cutting (Agbemenu et al., 2019). Interpersonal challenges are also reported, like feeling different and exposed in interactions with health care personnel unfamiliar with female genital cutting (Berggren et al., 2006). Women report working through pain (Perović et al., 2020), stigmatization during care (Chalmers & Omer-Hashi 2000, 2002; Jacobson et al., 2018), and sensing that their health care preferences are not valued in the same way as women without female genital cutting (Jacobson et al., 2018). It is important to become aware of the discourses that shape these experiences for women with female genital cutting because discourse, as conceptual knowledge, shapes the way people think about certain phenomena.

Discourse is communicated through text (i.e., newspapers and research articles) and verbal language (i.e., conversations; (DeVault & McCoy, 2006) M. DeVault & McCoy, 2006; Wetherell et al., 2003). People’s experiences can be shaped by the standards set through these mediums, which become taken up by lay people (Griffith & Smith, 1986). Often these standards are organized by professionals advocating against female genital cutting who may not have come from nor received training on the cultural contexts in which female genital cutting is practiced.

Looking to the terms used to refer to the practices of female genital cutting demonstrates how discourse may shape the popular imaginary; these terms include “circumcision,” “mutilation,” and “cutting.” Some founding anthropological work called it “circumcision” (Boddy, 1982; Gruenbaum, 1982; Hale, 1994; Shell-Duncan, 2001), corresponding to what the cultures themselves called the practices (Llamas, 2017; Ross et al., 2016). The importance of circumcision for both women and men has been highlighted by the Kipsigis (Kenya), whose name translates to “we the circumcised” (Daniels, 1970). Other words used by practicing communities to refer to the practices include: “sunna” (the way of the Prophet; Talle, 2008), “tahūr” (purification; Boddy, 2016), and “tahāra” (purification; Merli, 2008).

The term “mutilation” came into use in the west in the 1970s when second-wave feminist activists emphasized the practices’ harmful effects. Anti-female genital mutilation discourse began with the use of this term (Hosken, 1978, 1979), expanding to the human-rights view that the practices require eradication (Andro & Lesclingand, 2016). Hayes, 1975; Hosken, 1978, 1979 were some of the first researchers to use the term “mutilation.” Although they did not define their use of it, (Hosken, 1978) described “mutilation” as “an impediment … that can be prevented and eradicated much like any disease” (p. 155). A parallel discourse was thus induced, describing “mutilation” as a human-rights violation requiring eradication (Andro & Lesclingand, 2016). This human-rights focus was solidified at the 1995 Beijing Fourth World Conference on Women where there was a call to end the practices on those grounds (Boddy, 2007).

The World Health Organization (2008; 2018; 2020), United Nations Population Fund, and United Nations Children’s Fund (United Nations Population Fund & United Nations Children’s Fund, 2018) also call the practices “mutilation” in advocacy documents, citing intent for its eradication. This term is used to push policy changes toward what they consider as the protection of those affected by the practices. Organizations’ use of the term “mutilation” and subsequent notion that the practices should end displays how the use of discourse shapes the popular imaginary regarding female genital cutting.

Many researchers still use this naming (Abathun et al., 2016; Belihu et al., 2017; Isman et al., 2013), often to express the “sheer impact of the procedure” physically and psychologically (Dave et al., 2011, p. 103). However, researchers who aim to understand the social and bodily impact of the practices often call it “cutting” (Hearst & Molnar, 2013; Johansen & Ahmed, 2021; Johnsdotter, 2018; Johnson-Agbakwu et al., 2014). This term often describes the physical removal of genital nerve and muscle during the original procedure (Einstein, 2008; Jacobson et al., 2018; Perović et al., 2020). Similarly, Tostan (2018), an African organization known for their successful campaign to reduce female genital cutting, uses the term “cutting.” Compared to other terms, they consider this one as “less judgmental and value-laden.” For example, “circumcision” has been said to invite contrast to male circumcision (Gillespie, 2015, p. 1). “Mutilation” has been said to miss the practices’ nuances (Duivenbode & Padela, 2019), inviting defensiveness from those who do not view it as disfiguring (Gillespie, 2015). However, no term encapsulates the multiplicity of perspectives or complexity of the phenomenon.

Anti-female genital mutilation discourse is discussed as a “discourse” in few articles (La Barbera, 2009; Njambi, 2004), but is referred to using terms like “anti-female genital cutting/mutilation movement,” “anti-female genital mutilation interventions” (Østebø & Østebø, 2014), “anti-female genital cutting/mutilation law” (Boyle & Preves, 2000; Earp, 2014), and “anti-female genital cutting/mutilation activists” (Earp, 2014; Njambi, 2004). These terms are encapsulated within anti-female genital mutilation discourse to depict the people, strategies, and activities that set particular standards and work toward the goal of eradicating “mutilation.” It is important to note that anti-female genital mutilation discourse has targeted only genital cutting of those assigned *female* at birth, and not those assigned *male*. This highlights a double standard in many western countries for female genital cutting being viewed as “mutilation” and a human-rights issue, while male circumcision is generally accepted (though some scholars have challenged this double standard; see Earp, 2014).

While looking to the naming of the practices begins to demonstrate how related discourse shapes interpretation of the practices’ meaning, it is neither understood how women engage in such discourses, nor how they shape their care experiences. We conceptualize discourse as a social relation in the institutional ethnographic context in which this study was carried out.

### Language Used in this Article

We use varying terms to refer to the practices reflexively. We use “cutting” to describe the action without judging it or its practitioners. For women’s quotes, we mirror the language they used while critically examining organizations’ language. When describing the aforementioned discourse, we use the term “mutilation” to convey the terminology mobilized by institutions that perpetuate this discourse, which may have an impact on women’s care experiences.

We refer to the preparation that women with female genital cutting engaged in prior to health care encounters as “health work,” which draws attention to people’s activities in navigating care (Mykhalovskiy & McCoy, 2002). “Health work” is an extension of Dorothy E. Smith’s (creator of institutional ethnography, the approach to inquiry used in this article; Smith, 1987; 2005; 2006) generous notion of “work.” This attends to what people actually do, bringing the perspectives of everyday people into focus ((DeVault, 2006) Grace, 2019). For example, studies of individuals living with HIV found that they worked to make decisions and inform themselves about their condition and treatments (Mykhalovskiy & McCoy, 2002; Mykhalovskiy et al., 2004). Often, people do not recognize their activities as “work” despite the effort involved (Smith, 1987).

We also refer to reproductive health care encounters as any appointment involving some aspect of obstetric/gynecological care administered by either a general practitioner or by an obstetrician/gynecologist. This ranges from vaginal examinations to deinfibulation (a surgical technique to detach labia previously sewn together).

## Methods

### Entering into the Research

We originally undertook this research when we heard from women with female genital cutting at local community meetings in Toronto of their troubling reproductive health care encounters. We tied this with what had been described in the literature (Belihu et al., 2017; Berggren et al., 2006; Chalmers & Omer-Hashi 2000, 2002; Jacobson et al., 2018; Johnson et al., 2005; Moxey & Jones, 2016). Both sources indicated that women have felt stigmatized and treated differently from women without female genital cutting. We did not originally enter into the research aiming to investigate any particular discourse but inductively looked for traces of such social and discursive relations. When traces of anti-female genital mutilation discourse were noticed in participants’ language, we then began to inquire into this.

We recognize our position as academics studying this topic (in public health, anthropology and psychology), and as western-born, white individuals, rather than members of the community affected by female genital cutting, who are often racialized. DJ, who conducted interviews and wrote the first draft of this article, used the Social Identity Map to practice positionality (see Jacobson & Mustafa, 2019). DJ reflected on her privilege and social position as an outsider to Black women with female genital cutting who face unique oppression based on their intersecting identities and whose standpoints provided entry into this work (Bowleg, 2012). Particular nuances may have been missed in our interpretation of the data due to our lack of direct experience with the practices and of being racialized in the health care system. An advisory team of women with female genital cutting in Toronto were consulted to ensure that our approach was considerate, culturally appropriate, and of value. Entering into the research, it was a priority to make space for the perspectives of Black African immigrant women with female genital cutting.

### Institutional Ethnography

We used institutional ethnography under the critical social paradigm to pursue non-exploitive research with a social justice orientation (Campbell & Gregor, 2002). With institutional ethnography, the goal is to reveal how people’s experiences come to happen. Ontologically, we view reality as historically embedded in the ways individuals and institutions function (Ramazanoglu & Holland, 2011; Smith, 2006). We have employed the epistemological shift with institutional ethnography that centers knowledge production on empirically tracing people’s difficult experiences within the institutions they traverse, rather than looking to the people themselves (Deveau, 2009; Sinding, 2010). This influenced our approach to begin by understanding participants’ accounts of difficulty with health care via interviews before looking beyond any one account to link these experiences with what happened at the institutional level. One influence in the development of institutional ethnography has been Foucault’s use of discourse (Foucault, 1980). This influence guided our pursuit to understand how discursive knowledge informs, constrains, and shapes the way that people think about and experience particular phenomena (Wetherell et al., 2003).

### Recruitment

This study was approved by the University of Toronto research ethics board and was guided by advisors from the community (two women with type IIIb female genital cutting and two experts in female genital cutting research). Advisors provided insight into the research’s value and direction (i.e., approach to interviews/questions, the study’s value for the community).

Recruitment lasted from June 2019 to March 2020. We posted flyers at libraries, community centers, and universities, which was not a fruitful method. We instead recruited with the help of a community health center and *via* referral. Participants reached out to DJ, indicating interest in the study, or DJ reached out to referred women who agreed to have their information shared.

### Participants

Inclusion criteria were to (1) have type IIIb female genital cutting; (2) be 18 years of age or older; and (3) have accessed reproductive health care in Toronto no more than 10 years prior to the interview. It was determined that women had female genital cutting through self-report. Recruitment was restricted to women with type IIIb female genital cutting because it is the most severe form. Recruitment ended when we had garnered an in-depth understanding of how participants’ care interaction took place within the broader setting of the reproductive health care system. Participants were compensated with $40 CAD.

Eight women aged 25–70 from Sudan, the Gambia, Somalia, and Kenya participated. They had female genital cutting between the ages of 4–7, immigrated to Canada between 1993–2019, and had education ranging from high school to a master’s degree. Seven had between one–four children and one had no children. Women sought care for childbirth, routine tests, and ailments (i.e., pain). Each woman’s background is not provided to protect privacy.

### Interviews

One-on-one, audio-recorded, in-depth, semi-structured, and qualitative interviews were conducted with eight women with female genital cutting. Open-ended questions invited women to share their knowledge and perspectives. Women were prompted to describe their reproductive health care experiences with questions such as: “Have you ever been referred to a reproductive health care specialist?” and “How was that experience for you?” Participants’ expertise in their lives was prompted by asking about recurring events and words repeatedly used. This began to reveal the pattern of how the health care encounter happened (Campbell & Gregor, 2002). We wanted to understand women’s knowledge, how they put it together, what went on during the reproductive health care encounter, and how it was managed (DeVault & McCoy, 2006).

Interviews were 1–2 hours long and held where women were most comfortable, as long as it was private (University of Toronto, over the phone, community health center). Participants completed written consent and socio-demographic forms and chose a pseudonym by which they are referred in this article. We described that the study was about understanding the reproductive health care interaction and how women’s experiences were shaped. An interpreter, briefed on anonymity, confidentiality, and the study’s purpose, was recruited for two women who preferred to speak a language other than English. Interviewees were not obliged to answer any questions and could withdraw at any time from the study without penalty, though none withdrew.

### Analysis

Analytical thinking began during interviews by checking/re-checking with participants our understanding of what happened surrounding their care encounters (Campbell & Gregor, 2002). Interviews were transcribed and read critically multiple times. DJ began to notice particular work practices across participants’ accounts, including their preparation prior to entering the clinic. DJ then used indexing as an analytical tool by grouping together instances of work across accounts (Rankin, 2017). For example, when participants mentioned something they did that took effort (i.e., finding a doctor/clinic), an index was created (i.e., “work of finding a doctor/clinic”). Then instances of this work across participants’ accounts were grouped to one organizational location using NVivo, allowing us to link these everyday accounts to their greater social organization. Co-authors weighed in on indexes, which were then further developed through analytic writing.

## Results

The two main findings of this article are that participants engaged in anti-female genital mutilation discourse and also did “emotional health work” to avoid anticipated trauma in the health care context based on the stigmatization they felt in Canadian society. We refer to this work as “emotional health work” because to get it done, participants had to deal with emotions like fear, worry, shame, and courage. We first present the particular language and frames participants took up that were reminiscent of this discourse, which condemned female genital cutting as a violation of human-rights. We then connect the ways that this discourse shaped participants’ emotional health work to prepare for their care encounters.

### Anti-Female Genital Mutilation Discourse in Participants’ Language and Accounts

In this section, we demonstrate the traces of anti-female genital mutilation discourse found in participants’ language. We then discuss the ways in which this discourse both worked to protect the rights of women and girls with female genital cutting, but also stigmatized them.

*Use of the term “mutilation” for human-rights advocacy.* Traces of anti-female genital mutilation discourse were observed in participants’ language through the use of the term “mutilation” itself. One participant said, “I call it female genital mutilation … It’s like somebody cut off my nose, it’s a part of my body!” Participants made clear that the human-rights goal of eradicating the practices—an aim perpetuated by those who mobilize anti-female genital mutilation discourse—was a top priority. Another woman stated, “These are healthy, functional organs that are being cut. So, it is mutilation … This is human-rights. Organs are cut for non-medical reasons.”

Women came to use the term “mutilation” after exposure to educational materials in natal countries (where women were born). These would have been read privately in the natal context because abolition of female genital cutting was seen as an inappropriate topic. Through higher education, women were exposed to materials that framed the practices as “mutilation.” One participant explained: “Education played a huge part in my understanding or critical consciousness of female genital mutilation … the impact it has on the sexual and reproductive health rights of girls and women.” Therefore, the language of anti-female genital mutilation discourse, and particularly the use of the term “mutilation,” allowed participants to advocate for their own rights as well as those of other women and girls.

Although this discourse helped participants advocate against the practices, women also realized that it originated by westerners without female genital cutting. One participant said, “It has different names in [natal country]. We say ‘circumcision’. This ‘female genital mutilation’ … is terminology given on this process from people outside.” Highlighted was the split between the way female genital cutting was viewed and named in practicing and non-practicing, western countries. Another woman also saw the discourse as outside her culture, but strategically used western terms for political advocacy: “If I were talking to women from my background … we call the name people call it locally … When it comes to English, personally, I like ‘female genital mutilation’. I don’t like getting out of the realm of human-rights.” The term “mutilation” therefore allowed participants to advocate for “human-rights,” but also distinguished women with female genital cutting from those “outside” who were not “mutilated.”

*Duality of anti-female genital mutilation discourse: A vehicle for advocacy and stigmatization*. Although anti-female genital mutilation discourse helped women advocate for the protection of those affected by the practices, it also worked to stigmatize them. One participant saw how the language of the discourse perpetuated a societal view of female genital cutting as “bad”: “The way they put it … western, they say it [the practice] is bad, whoever does it is bad, whoever it is done to is bad, the culture is bad, everything is bad.” This participant explained how the media’s use of anti-female genital mutilation language was a source of stigmatization because newspapers “label people” and “talk about the barbaric thing … how the woman is not complete, they talk so many negative things … And the women hear it!”

Participants were concerned about the media’s lack of understanding of female genital cutting and were made to feel different because of portrayals perpetuating anti-female genital mutilation discourse. One participant said: “Sometimes, you hear or read … it’s not something they understand how it happens … I don’t even remember the particular report where I read that, but I remember that concern.” Another woman explained how media portrayals made women feel attacked: “That affects us, because we are human beings, we have feelings … women feel attacked. And the result is not a good one.” An additional participant said, “You feel bad about yourself. Okay, am I different from other people? … But we can’t change that … I just have to accept it—even though I’m different.” It is thus not surprising that participants constructed a particular narrative regarding the practice; specifically, that they felt different and stigmatized because of their female genital cutting.

Participants further described that when people read these articles, they pick up on the anti-female genital mutilation language and sentiment, absorbing and reproducing stigmatizing ideas:

Non-circumcised, other people, when they read that, they think that this is bad—which I agree. But, [they think] people who have been done to are bad too. They put all in one category … A non-circumcised person reads the newspaper. And they will say, “Oh my goodness! It’s barbaric!” … When a group of non-circumcised people read the newspaper that talks negatively … they attack the whole culture and especially women.

Media articles’ use of anti-female genital mutilation language and sentiment, and readers of such news articles absorbing and perpetuating this othering of women with female genital cutting, separated practicing cultures from unmentioned but imagined non-practicing, “non-barbaric” cultures. Anti-female genital mutilation discourse thus presents a paradox: those who engage in it aim to protect women and girls, but the language and sentiment used also leads to stigmatization.

### Anti-Female Genital Mutilation Discourse Shapes Participants’ Emotional Health Work

In this section, we connect the ways in which learning that they were stigmatized through their engagement in anti-female genital mutilation discourse motivated participants to engage in emotional health work to prepare for their health care encounters.

Participants feared the stigmatization that was perpetuated through anti-female genital mutilation discourse being extended into their reproductive health care. One woman explained, “I think a lot about stigma associated to it [the practice] and how I would be treated as a person [in health care].” The fear of this stigmatization carrying into their care encounters motivated women to engage in two forms of work to prepare for these interactions, which are discussed in this section: Learning about family and friends’ previous care experiences and finding a doctor who was either familiar with female genital cutting or respectful of racialized communities.

We refer to this effort as “emotional health work” because of the emotions that participants had to manage while engaging in this work. For example, one participant (who previously described feeling stigmatized by anti-female genital mutilation language and sentiment in news articles) worried that her doctor may embody this discrimination: “Shame is in the back of my mind. The doctor may think, ‘Why did she accept that?’ He might undervalue me! That goes through every woman’s mind when seeing a doctor—will he treat you the way he treats non-circumcised women?” This required participants to have courage as they sought care. Through anti-female genital mutilation discourse, the health care system reached beyond and before the clinical encounter, extending the institutional and temporal bounds of the appointment.

*Learning from family and friends’ experiences: “Everybody has to know from the other.”* In response to their stigmatization in the media and society more broadly, participants engaged in emotional health work by learning from their community about previous health care encounters. One participant explained: “I have friends from my country here. They told me about [their experience] … Everybody has to know from the other. … I hear sometimes [about what will happen] … But, before they talk about it, you don’t understand anything.” This participant described the emotional component of this work as she managed fear about what might happen at her own appointment: “I was scared to know how it [the appointment] was going to happen.”

Another participant (who was experienced with health care) described how women with little health care experience in Canada sought advice from more experienced women: “If a woman asks me, ‘I’m seeing a doctor. What should I say? What should I expect?’ we talk about that. From the woman’s perspective, she has to be prepared when she is going to the doctor.” The notion that women must “be prepared” underscores how participants viewed the stigmatization perpetuated by anti-female genital mutilation language and sentiment as having permeated into the health care system. Thus, they had to work to prepare themselves and others for the encounter.

Participants learned that their family and friends had experienced unwanted gynecological procedures. One participant heard from friends that “there are a lot of rushed decisions to C-section.” As another woman put it, “They usually do [episiotomy] for so many people, so we hear about it.” This particular woman managed fear as she contemplated these unwanted procedures as posing danger to her in the health care context: “I asked many people … I guess I am scared because … I know that cutting is very dangerous.” Female genital cutting is often interpreted in western health care contexts as causing a danger to women so unwanted procedures are undertaken to avert that danger. However, women viewed these unwanted procedures as actually being the danger to them.

The biomedical literature confirms that women with female genital cutting are more likely than western-born women without it to undergo C-section (Johnson et al., 2005) and episiotomy (Belihu et al., 2017). Although participants did not necessarily use biomedical literature as a knowledge source, they did engage in the complexities of anti-female genital mutilation discourse as evidenced by their reproduction of the language and frames. They learned that stigmatization (resulting from this discourse) made its way into health care as it influenced the unwanted procedures they may have to undergo.

One participant also heard from family members that “health care is still not ready for this situation” and that “[doctors] don’t learn any disease of Black people.” This participant described being stigmatized at different intersections, as the health care system was not prepared to care for ailments prominent in Black women and women with female genital cutting: “Although we have a diverse society, diseases of certain communities are not given any importance. That is upsetting.”

Learning from family and friends that the health care system was underprepared to care for bodies different from the western norm and reflecting on the stigmatization and unwanted treatment they might face, emotions arose for women about what might happen during their care encounter. Participants described the fear of “feeling insecure” and “worthless” during their appointments. They also described managing worry about doctors’ knowledge of and ability to treat them. While one participant said, “The doctors, they don’t know about this! Nothing!”, another woman said, “Doctors may have never seen mutilation … Some may think it is a deformation … The worry was, is she in a position to know female genital mutilation.”

An additional participant said how it took “a lot of courage” and preparation to face a health care system that she worried was not prepared to care for her: “Am I going to talk to someone who doesn’t know this practice? Who wouldn’t know how it feels? Who may not have a deep understanding? I don’t know if my culture would be judged. Or if I would be judged.” Becoming aware that stigmatization perpetuated through anti-female genital mutilation language and sentiment had made its way into their family and friends’ health care encounters raised these emotions.

*Work to find a doctor/clinic: “If I could have someone with an open mind.”* From engaging in anti-female genital mutilation discourse and learning that their stigmatization carried into reproductive health care, participants became motivated to mitigate potential discrimination by finding a doctor and/or clinic that could best understand their bodies. Women sought doctors with whom family and friends had favorable experiences. One participant, who discussed the danger of having female genital cutting in the western health care context, became motivated to consult her community for referral to a “good” doctor: “I was lucky because I found this doctor. She is really good. She understands … I have many friends from my country here. They told me about her.”

The learning relationship was reciprocal. This same participant also described an obligation to tell her community about positive experiences to continue the chain of referral to that same doctor: “In our community, they talk about everything. If you find a good doctor, you have to tell this … If somebody is feeling anything [symptoms], you have to tell them, ‘You should go for this [doctor].’” Sharing knowledge was critical to avoiding stigmatization and unwanted treatments.

When “good” doctors were not accessible, referrals to clinics known to be respectful of racialized communities were sought. This highlights women’s dual identities as having female genital cutting and being Black. One participant did not know anyone to consult when she arrived in Toronto, so she engaged in emotional health work by managing her fear when she asked new, uncut friends for a recommendation: “I was linked to this organization through my friends … I wanted to go but I was scared. [I asked for] a feminist organization that deals with racialized communities, that will understand my position and respect my personhood.” This participant wanted a doctor who could respond well to one of these dual identities. When she contacted the recommended clinic, she asked the receptionist to recommend a doctor who could best respond to her: “I know my reality. It [female genital mutilation] might not be the experience of anybody … but if I could have someone with an open mind.” This participant knew she was viewed as different in Canada—a view reinforced by anti-female genital mutilation discourse. Her fear of being treated by someone who did not understand these realities resulted in her work to seek out a doctor with an “open mind.”

## Discussion

This study explored the social relations that shaped women with female genital cutting’s reproductive health care experiences. We found that anti-female genital mutilation discourse and women’s engagement in it shaped the work they did prior to the care encounter. Participants’ fear of experiencing discrimination in their care encounter due to the media’s anti-female genital mutilation discourse, as well as learning that community members faced stigmatization during their care all led to extensive preparatory work.

### Emotional Health Work and the Navigation of Health Care

Despite participants not being raised in the Canadian health care system, they were savvy in their navigation of care. Part of the expression of this savvy was the expertise that participants showed *via* their language and knowledge of reproductive health care. They proactively sought and shared experiential knowledge with their community, were educated and familiar with anti-female genital mutilation language, and grasped how doctors often approached female genital cutting, taking steps to mitigate this for future medical appointments.

Although the steps participants took to ensure a successful health care encounter could be called “health work” (Mykhalovskiy & McCoy, 2002; Mykhalovskiy et al., 2004), the emotions involved went beyond this notion. Emotions are seldom discussed in the literature in terms other than psychological trauma (Knipscheer et al., 2015; Mulongo et al., 2014; Vloeberghs et al., 2012). However, participants’ work aimed to *avoid* the trauma they anticipated based on how female genital cutting is viewed in the Canadian popular imaginary. Emotional health work was not about certain emotions or emotional experiences, but centered around women’s navigation of emotional hurdles to get their preparatory work done.

Previous research has explored the role of emotions in health care satisfaction (Helena Vinagre & Neves, 2008) and managing patients’ emotions (McColl-Kennedy et al., 2017). However, these have focused on health care personnel’s navigation of emotions rather than patients’. Other work has centered on cancer patients’ emotional expression (Porter et al., 2005), minority patients’ emotional cues (Schouten & Schinkel, 2015), and the role of emotions in the organization of care (Mark, 2005). However, these articles did not discuss such emotions as *work*. Attending to emotional health work is vital as emotions are implicated in health care and often influence the onset and progression of diseases (i.e., rheumatoid arthritis; Solomon & Moos, 1964).

Future investigation of emotional health work may begin with people subject to gendered practices or racial disparities. For example, a man with a lump in his breast might also engage in emotional health work to navigate the health care system in which breast cancer is traditionally thought of as a “woman’s disease” (Midding et al., 2018, p. 2194; Skop et al., 2018). A Black woman giving birth may also engage in emotional health work to navigate the health care system in which Black infants are subject to higher mortality rates than white infants (Matthews et al., 2015; Wallace et al., 2017). The work people do to maintain emotional health prior to the care encounter is therefore an overlooked area ripe for further research that might aid in understanding the link between emotions and health.

### Stigmatization and Anti-Female Genital Mutilation Discourse

Participants were acutely familiar with the human-rights language of anti-female genital mutilation discourse. Discourse can be circulated in multimodal ways beyond written/unwritten texts (Han, 2015). However, due to our use of institutional ethnography, which underscores connections between people and texts (Grace, 2013; Rankin, 2017), we attended to this medium.

Women in this study pointed to the language of anti-female genital mutilation discourse in attention-grabbing news articles that have referred to the practices as “abhorrent” (Mordaunt, 2020, The Times), “barbaric” (Hussain, 2017; The Blade) and “a horrific nightmare” (CBC Radio, 2016). This discourse has been identified as problematic for women with female genital cutting. For example, the term “barbaric” can be linked to the racist perception of “primitive” African cultures—a stigmatizing view originating in the west (Manderson, 2004, p. 287; Njambi, 2004).

Discriminating language used by the media can also be spread in the comments section of online news articles. One commenter said, “These barbarians don’t want females to enjoy sex.” Another wrote in a reply: “Obviously you’re not familiar with how horrifying female genital mutilation is” (Allam, 2018; Buzzfeed News). The stigmatizing language and ideas of anti-female genital mutilation discourse become absorbed and perpetuated by everyday people who repeat the media’s language, revealing the public’s learned negative opinions–an occurrence which participants were all too well aware.

This discourse may have increased participants’ feeling of unbelonging, which was exacerbated by their own reference to the practice as “mutilation”—all contributing to their preparatory emotional health work. In another Canadian study, women with female genital cutting viewed the practices as “normal” in their natal community. However, in Canada, they too experienced a sense of unbelonging, questioning how “normal” they were (Jacobson et al., 2018). In the current study, participants’ stigmatization separated them from Canadian-born women without female genital cutting and led them to describe their bodies as partial and violently disfigured. Although participants were allied with the anti-female genital mutilation discourse goal of protecting those affected by the practices, they also recognized how this discourse stigmatized them.

### Recognizing Racism

Anti-female genital mutilation discourse is part and parcel of the broader racial discrimination “built into forms of social organization … rendered invisible through repetition and routinization” (Hamed et al., 2020, p. 1662). This was demonstrated by the media’s ubiquitous defamation of women with female genital cutting, and lay people’s repetition of such terms. Ironically, the use of the term “mutilation” was originally intended to advocate for women’s rights by activists of the second-wave feminist movement (Hosken, 1978, 1979). However, the movement was critiqued for being “white led,” for focusing on sexism as the “ultimate oppression,” and continuing marginalization of the “activism and world views of women of colour” (Combahee River Collective Statement, 1983; Thompson, 2002, p. 337). To this day, female genital cutting advocacy is still often rooted in the fight for gender equity (World Health Organization, 2008) and rarely in efforts against anti-Black racism (and racism of people of colour).

Despite this start to recognizing how historically women came to be helped and hindered by anti-female genital mutilation discourse, there is work needed beyond the scope of this article to study the link of racism and advocacy within the term “mutilation.” For example, even though the World Health Organization is purported to have updated its language to call the practices “mutilation/cutting” (Boddy, 2016), highly cited facts pages still use “mutilation” (World Health Organization, 2018, 2020). The addition of the “C” also might not break down the term’s stigmatizing effects. It is pertinent for organizations to take up anti-racist frameworks with the goal of working toward health equity to address women’s safety, effects of the practices, and the consequences women face as a result of racism embedded in anti-female genital mutilation discourse.

Participants in the current study recognized this racism through their comments on the absence of Black professionals and information on Black populations in the reproductive health care context. This highlights the urgent need, already cited in the literature, for representation of diversity (Flores & Combs, 2013; Nair & Adetayo, 2019), as well as a focus on cultural competency (Johnson-Agbakwu et al., 2020; Johnson-Agbakwu & Manin, 2021) and structural determinants of health/health inequities (Crear-Perry et al., 2021) in health care education and delivery. Making space in professional roles for representation and implementing training that combats “racism, implicit bias, and microaggressions at the institutional as well as individual level” are only a few of many necessary components to work toward improved health care treatment of minority populations including women with female genital cutting (Johnson-Agbakwu et al., 2020, p. 2). Examples have been outlined of important actionable steps to address structural racism (Johnson-Agbakwu et al., 2020) and practical considerations for clinicians and researchers who work with women with female genital cutting (Johnson-Agbakwu & Manin, 2021) that will be critical to consider within the circle of health care for women with female genital cutting.

## Conclusion

We explored the social relations that shaped women with female genital cutting’s reproductive health care experiences. Anti-female genital mutilation discourse, which influences public conversations through stigmatizing language by non-governmental agencies and the media, shaped the emotional health work that women engaged in prior to appointments. This discourse had a dual effect, both helping participants to advocate against the practices and hindering them through a stigmatizing framework for the practices, women, and cultures. This prompted participants to work on mitigating anticipated adversity through practical and emotional preparation for their health care encounter.

Part of this preparation was engaging with anti-female genital mutilation discourse, which both helped and hindered women with female genital cutting. It is vital to question existing human-rights and advocacy discourse that leads to measures that stigmatize those who are meant to be protected. By making space for the perspectives of Black women with female genital cutting in western reproductive health care contexts and in the strategies that aim to protect them, we can work toward assuring expert and inclusive care in which women might not have to work so hard before even entering the four walls of the clinic.
